# Xanthotoxin reverses Parkinson’s disease-like symptoms in zebrafish larvae and mice models: a comparative study

**DOI:** 10.1007/s43440-020-00136-9

**Published:** 2020-07-22

**Authors:** Ewelina Kozioł, Krystyna Skalicka-Woźniak, Agnieszka Michalak, Katarzyna Kaszubska, Barbara Budzyńska

**Affiliations:** 1 Independent Laboratory of Natural Products Chemistry, Department of Pharmacognosy, Medical University of Lublin, Lublin, Poland; 2 Laboratory of Behavioral Studies, Chair and Department of Medical Chemistry, Medical University of Lublin, Lublin, Poland; 3 Department of Pharmacology and Pharmacodynamics, Medical University of Lublin, Lublin, Poland

**Keywords:** Parkinson’s disease, Coumarin, Zebrafish, Mice, 1-Methyl-4-phenyl-1,2,3,6-tetrahydropyridine, 6-Hydroxydopamine

## Abstract

**Background:**

The aim of this study is to preliminary evaluate the antiparkinsonian activity of furanocoumarin—xanthotoxin, in two behavioral animal models, zebrafish larvae treated with 6-hydroxydopamine and mice treated with 1-methyl-4-phenyl-1,2,3,6-tetrahydropyridine in order to compare both models.

**Methods:**

Xanthotoxin was isolated from *Pastinaca sativa* L. (*Apiaceae*) fruits. Then, the compound was administered by immersion to zebrafish 5 days after fertilization (dpf) larvae or intraperitoneally to male Swiss mice, as a potential therapeutic agent against locomotor impairments.

**Results:**

Acute xanthotoxin administration at the concentration of 7.5 µM reversed locomotor activity impairments in 5-dpf zebrafish larvae. In mice model, acute xanthotoxin administration alleviated movement impairments at the concentration of 25 mg/kg.

**Conclusions:**

The similar activity of the same substance in two different animal models indicates their compatibility and proves the potential of in vivo bioassays based on zebrafish models. Results of our study indicate that xanthotoxin may be considered as a potential lead compound in the discovery of antiparkinsonian drugs.

## Introduction

Parkinson’s disease (PD) is a chronic central nervous system (CNS) disease with characteristic pathological changes including loss of dopamine-secreting neurons within substantia nigra and the presence of Lewy bodies. Main manifestations of the disease cover motor function decline, e.g., tremor, bradykinesia, postural instability; non-motor symptoms, e.g. nausea, depression, dementia, sialorrhea; as well as cognitive impairment [1, 2]. Since 1817, when PD had been described by James Parkinson for the first time, an effective treatment has not been proposed. A few therapeutic strategies are used in the treatment of PD: Levodopa (l-DOPA) and dopamine agonists activating dopamine receptors, monoamine oxidase type B (MAO-B) inhibitors and catechol-*O*-methyltransferase (COMT) inhibitors blocking dopamine degradation, or *N*-methyl-d-aspartate(NMDA) receptors and acetylcholine (ACh) receptors antagonists [1].

8-Methoxypsoralen known also as methoxsalen or xanthotoxin (XT) is a natural furanocoumarin occurring in many plants belonging to the *Apiaceae* family. It was found in 1911 in an alcoholic extract of *Fagara zanthoxyloides* Lam. The structure of the compound was determined in 1936 and in the same year, it was reproduced synthetically [3]. Since that time, the interest in XT for its pharmacological action has been increasing. In 1974, Parish et al. described the use of XT in psoralen and ultraviolet A radiation therapy (PUVA) of psoriasis, which is a combination of orally administrated psoralen derivatives and exposure to UVA radiation [4]. Today, XT is a drug used for the treatment of psoriasis and vitiligo [5]. In recent studies, XT was found to be an interesting agent acting on the CNS, enhancing the memory acquisition and consolidation and reversing memory impairment in mice [6, 7]. After systematic administration of XT occurred inhibition of AChE in both hippocampus and prefrontal cortex, which proves that this coumarin crosses the blood–brain barrier after systemic administration [8]. XT and related compounds showed protective properties against tonic–clonic seizures; additionally, XT enhanced the anticonvulsant effect of conventional drugs like carbamazepine, sodium valproate or phenobarbital and increased their concentrations in the brain [9, 10]. XT showed also moderate neuroprotective activity against glutamate-induced toxicity [11].

The most commonly used animal model of PD developing syndromes of the disease is based on the administration of neurotoxin, 1-methyl-4-phenyl-1,2,3,6-tetrahydropyridine (MPTP). MPTP is metabolized to 1-methyl-4-phenylpyridinium (MPP +), which is a radical interfering with the mitochondrial respiratory chain of neural cells. As a consequence of mitochondrial dysfunction, this neurotoxin induces the loss of dopaminergic neurons in the substantia nigra [12, 13].

Another model of PD in rodents, as well as zebrafish, involves the induction of symptoms of the disease using 6-hydroxydopamine (6-OHDA), a neurotoxin selectively destroying catecholaminergic neurons including the nigrostriatal system. Pathophysiological changes in nigrostriatal neurons are similar to those caused by MPTP and, in both models, the participation of oxidative stress is firmly established [13–15].

The aim of the study was to compare effects of XT on motor functions in zebrafish and mice, applying different models of PD, but with similar molecular mechanism. For this reason, two models of neurodegeneration in the substantia nigra were used: treatment with 6-hydroxydopamine (6-OHDA) in zebrafish and with 1-methyl-4-phenyl-1,2,3,6-tetrahydropyridine (MPTP) in mice. This is the first and preliminary study of potential antiparkinsonian activity of XT in PD models.

## Materials and methods

### Animals and treatment

#### Zebrafish

*Danio rerio* stocks of the AB strain were maintained at 28.5 °C, on a 14-/10-h light/dark cycle under standard aquaculture conditions, and fertilized eggs were collected via natural spawning. Embryos were reared under standard light/dark cycle in embryo medium: pH 7.1–7.3, 17.4-µM NaCl, 0.21-µM KCl, 0.12-µM MgSO_4_ and 0.18-µM Ca(NO_3_)_2_ in an incubator at 28.5 °C. To the procedure, 5 days post fertilization (dpf) larvae (total number 48, 24 per group) were used, and all experiments were completed before 120-h post fertilization, before larvae start feeding independently. According to EU Directive, 2010/63/EU, there is no need of local ethics committee approval for larvae on this stage of development. Immediately after the experiment, larvae were killed by immersion in 15-μM tricaine solution.

#### Mice

Naive male Swiss mice (total number 48, 8 per group), 6 weeks old (25–30 g), delivered from the Centre of Experimental Medicine, Medical University of Lublin, Poland and kept under standard laboratory conditions (12-h light/dark cycle, room temperature 21 ± 1 °C, at least 1-week adaptation to the laboratory conditions) were used in experiments. Animals had free access to tap water and laboratory chow (Agropol, Poland). Animals were housed 4 per cage, in Individually Ventilated Cages (IVC)—Techniplast UK. Each experimental group consisted of 8 animals. All experiments were conducted in accordance with the National Institute of Health Guidelines for the Care and Use of Laboratory Animals and to the European Community Council Directive for the Care and Use of Laboratory Animals of 22 September 2010 (2010/63/EU) and were approved by the local ethics committee (63/2015). The experimenter was unaware of the animal’s group during experimentation. To minimize animal suffering, qualified and experienced experimenters handled the animals with utmost care. All animal experiments were performed between 9 a.m. and 2 p.m. Mice were subsequently euthanized with CO_2_.

### Drugs

XT was purified from dichloromethane extract of *Pastinaca sativa* L. (*Apiaceae*) fruits, collected in the Medical Plant Garden of the Department of Pharmacognosy, Medical University of Lublin (Poland). The location of the garden is as follows: Situation (position) 700 m N-W near Center of town Lublin, Latitude 51º 15′ 22″; Longitude 22º 33′ 51″; Altitude ca 185 m above sea level. Fruits were collected in the summer of 2014. The species was identified by specialists in botany—Mrs Krystyna Dąbrowska from Botanical Garden of Marie Curie University, Lublin and a voucher specimen (17/20) is kept in the Department of Pharmacognosy with the Medicinal Plant Unit.

Isolation was carried out by high-performance counter-current chromatography (HPCCC) with a two-phase solvent system composed of n-heptane, ethyl acetate, methanol and water with the ratio of 1:1:1:1 (v/v/v/v) according to a previously published method [16]. The purity of XT was 98.6% and was checked by HPLC. Structure determination was confirmed with NMR.

MPTP hydrochloride (M103) and 6-OHDA hydrobromide (162,957) were delivered by Sigma-Aldrich (St. Louis, MO, USA). MPTP was dissolved in a saline solution (0.9% NaCl) and 6-OHDA was dissolved in embryo medium. For mice experiments, XT was suspended in a 50 µL of 1% solution of Tween 80 and dissolved in a saline solution to achieve a concentration of 5 mg/kg. Tween 80 can be employed safely as a vehicle for neuropsychopharmacological experiments in doses not exceeding 1 ml/kg [17]. For zebrafish assay, XT was dissolved in DMSO (D8418, Sigma-Aldrich St. Louis, MO, USA) and diluted to proper concentration. The final concentration of DMSO in every sample was equal to 1%.

The doses of 6-OHDA (250 µM) was chosen based on the literature data [18]. The doses of XT in zebrafish study was chosen after determination of the maximum-tolerated concentration (7.5 µM) according to the previously described protocol [19] and were 1.5, 3, 5, 7.5 µM. The doses of MPTP (4 × 20 mg/kg, 1 day) was chosen based on literature data [20, 21]. The doses of XT in rodent study were chosen on the basis of our previous study [8, 22] and were 15 and 25 mg/kg.

### Behavioral tests

Locomotor activity in zebrafish larvae was measured using the dark chamber of an automated tracking device (ZebraBox system; Viewpoint, Lyon, France). Locomotor activity was calculated using ZebraLab software (Viewpoint, Lyon, France). The total distance moved was defined as the distance (in cm) that a larva moved during one 10-min session.

To evaluate the influence of XT on MPTP-induced hypokinesia, the Opto-Varimex-4 Auto-Track apparatus (Columbus Instruments, USA) was used. The locomotor cages are built from a transparent material (43 × 43 × 32 cm) with a lid. The cages are equipped with two rows of infrared emitters (each emitter has 16 laser beams), with detectors monitoring animal movements. Each mouse was placed individually into the cage for 30 min.

### Experimental design PD

#### The 6-OHDA-induced PD symptoms in *Danio rerio*

5 dpf larvae were allocated into groups (24 larvae per group) as follows:

(1) Group I: control (1% DMSO solution in embryo medium); (2) Group II: 6-OHDA control (250 µM); (3) Group III: XT (1.5 µM); (4) Group IV: XT (3.0 µM); (5) Group V: XT (5.0 µM); (6) Group VI: XT (7.5 µM); (7) Group VII: 6-OHDA (250 µM) + XT (1.5 µM); (8) Group VIII: 6-OHDA (250 µM) + XT (3.0 µM); (9) Group IX: 6-OHDA (250 µM) + XT (5.0 µM); (10) Group X: 6-OHDA (250 µM) + XT (7.5 µM).

**Fig. 1:**
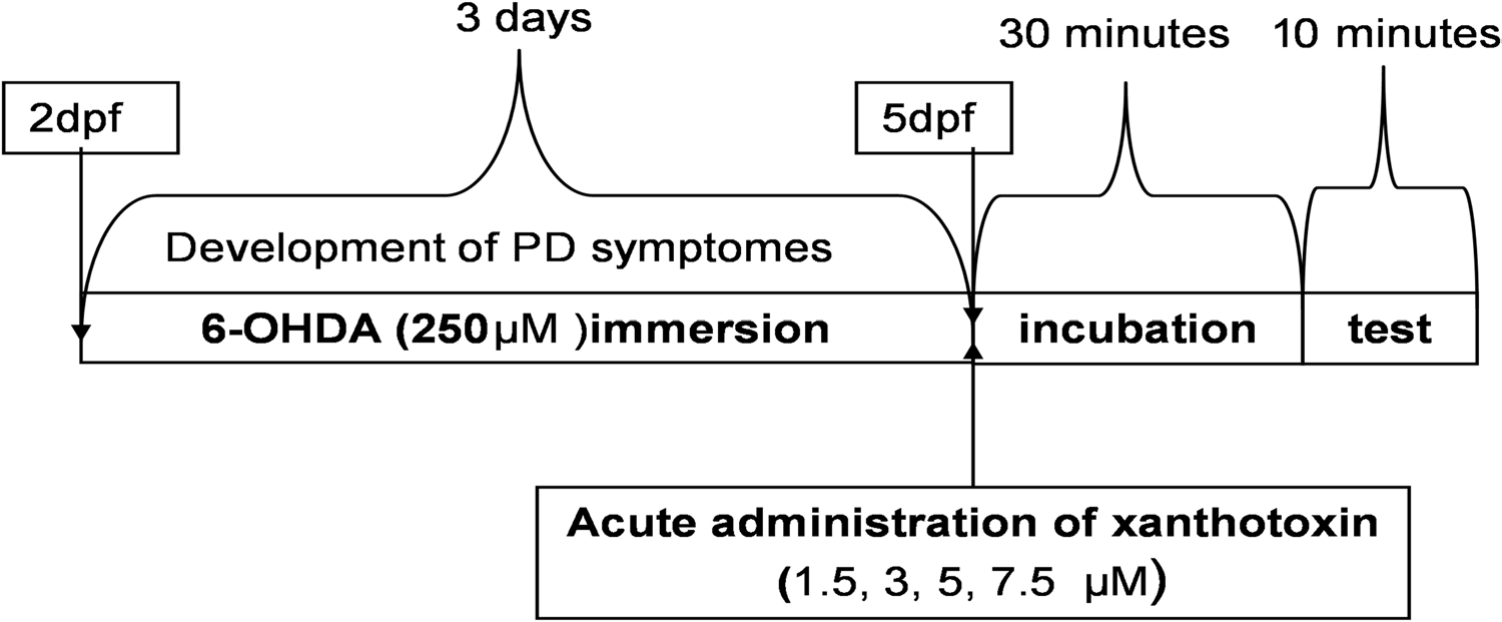
Diagram shows the schedule of administration of 6-hydroxydopamine (6-OHDA) (immersion) and xanthotoxin (XT) in zebrafish Parkinson’s disease protocol (*n* = 24)

The neurodegeneration in larvae was induced by maintaining zebrafish from 2 to 5 dpf in medium containing 6-OHDA in the concentration of 250 µM. The larvae were maintained in groups of 30 individuals. Three times a day, medium with neurotoxin was exchanged and a new concentration of 6-OHDA was prepared directly before administration. 5-dpf larvae were transferred to 96-well plates, 1 larva per each well, and different concentrations (1.5, 3, 5, 7.5, µM) of XT were administrated by immersion method. The incubation with drug lasted 30 min (Fig. 1).

### The MPTP-induced PD symptoms in mice

Mice were allocated into five groups, 8 mice per group: (1) Group I: control (saline, ip); (2) Group II: MPTP control (4 × 20 mg/kg × 1 day, ip); (3) Group III: XT (15 mg/kg; ip); (4) Group IV: XT (25 mg/kg; ip); (5) Group V: MPTP (4 × 20 mg/kg × 1 day, ip) + XT (15 mg/kg; ip); (6) Group VI: MPTP (4 × 20 mg/kg × 1 day, ip) + XT (25 mg/kg; ip).

**Fig. 2:**
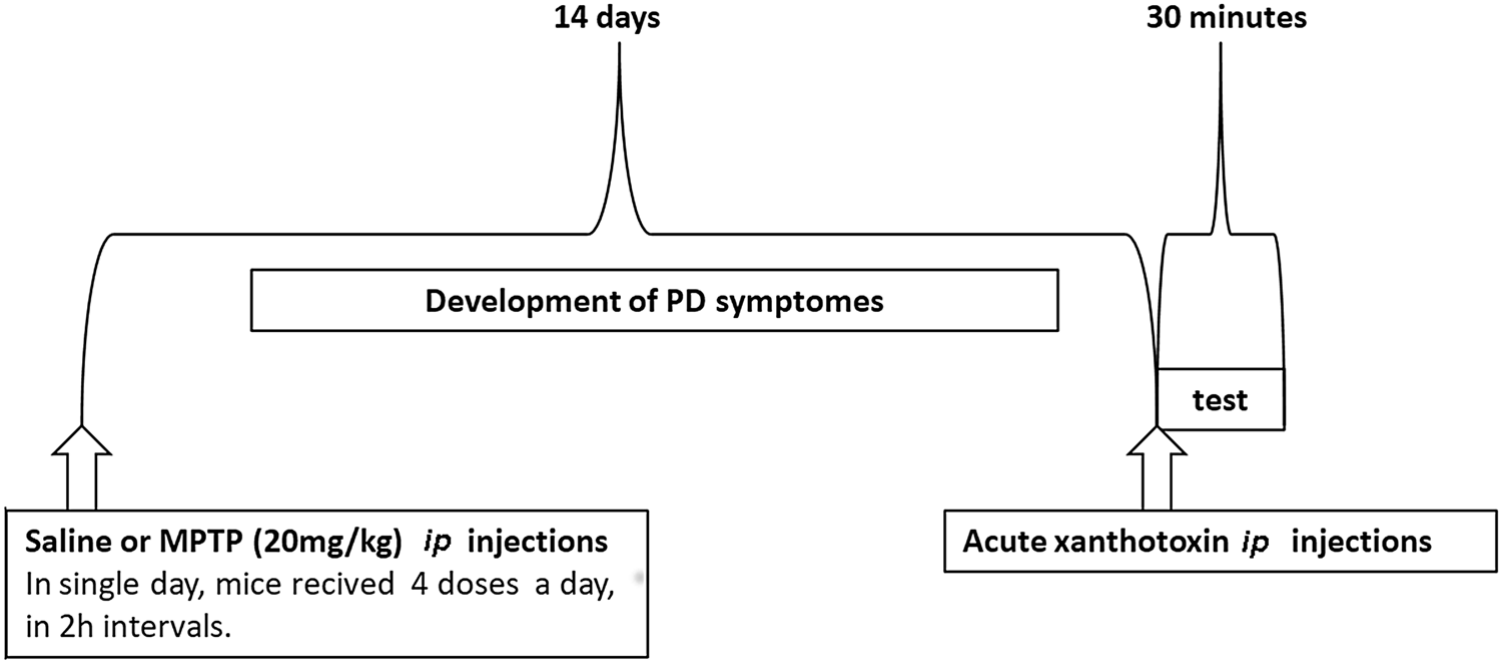
Diagram shows the schedule of administration of 1-methyl-4-phenyl-1,2,3,6-tetrahydropyridine (MPTP, ip) and xanthotoxin (XT) in mice Parkinson’s disease protocol (*n* = 8)

On the day of the experiment, mice received four ip injections of MPTP (20 mg/kg) in saline at 2-h intervals, 4 doses a day; control mice received saline only. 14 days after MPTP treatment, XT was administered acutely, each animal received a single XT injection, and then the locomotor activity test was performed (Fig. 2).

### Statistical analysis

Two-way ANOVA was used to perform the statistical analyses. Bonferroni’s post hoc test was calculated when appropriate. When the confidence limit was calculated as *p* < 0.05, the results were considered as statistically significant. For statistical analysis, means (distance in cm traveled by mice) ± SEM were used. GraphPad Prism version 7.0 (GraphPad Software Inc, San Diego, CA, USA) was used for the data analysis and for graphics.

## Results

### XT counteracts 6-OHDA-induced locomotor impairments

**Fig. 3:**
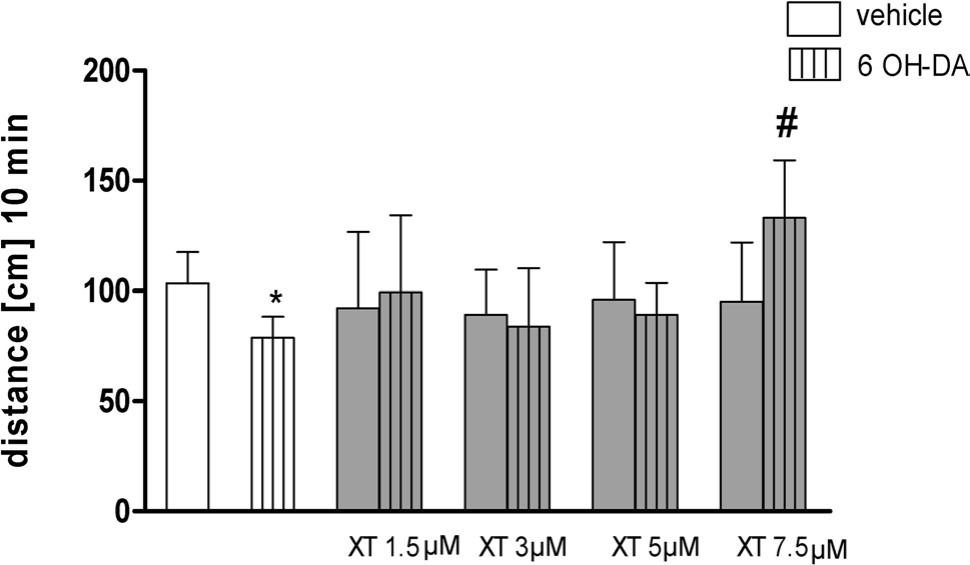
The effects of xanthotoxin (XT) on 6-hydroxydopamine(6-OHDA) induce locomotor impairments in zebrafish. Larvae were treated with E3 solution (vehicle) or 6-OHDA (250 µM, 4 days). 30 min before experiment larvae were incubated in different concentrations of XT (1.5, 3, 5, 7.5 µM). Data are presented as the mean ± SEM, *p < 0.05 vs. vehicle control group; ^**#**^*p* < 0.05, vs. 6-OHDA treated group, *n* = 24; Bonferroni’s post hoc test

Figure 3 shows the distance traveled by 5dpf zebrafish larvae after 6-OHDA and XT treatment (two-way ANOVA: pretreatment (saline/MPTP) *F*_4,144_ = 15.81, *p* = 0.0001; treatment (saline/coumarins) *F*_5,144_ = 25.45, *p* < 0.001; and interaction *F*_5,144_ = 21.28, *p* < 0.001. Treatment with 6-OHDA significantly decreased distance traveled by larvae as compared to 1% DMSO control group, *p* < 0.05. Acute administration of XT at the concentration of 7.5 µM reversed locomotor activity impairment induced by 6-OHDA, *p* < 0.05 (Fig. 3).

### XT counteracts MPTP-induced locomotor impairments

**Fig. 4:**
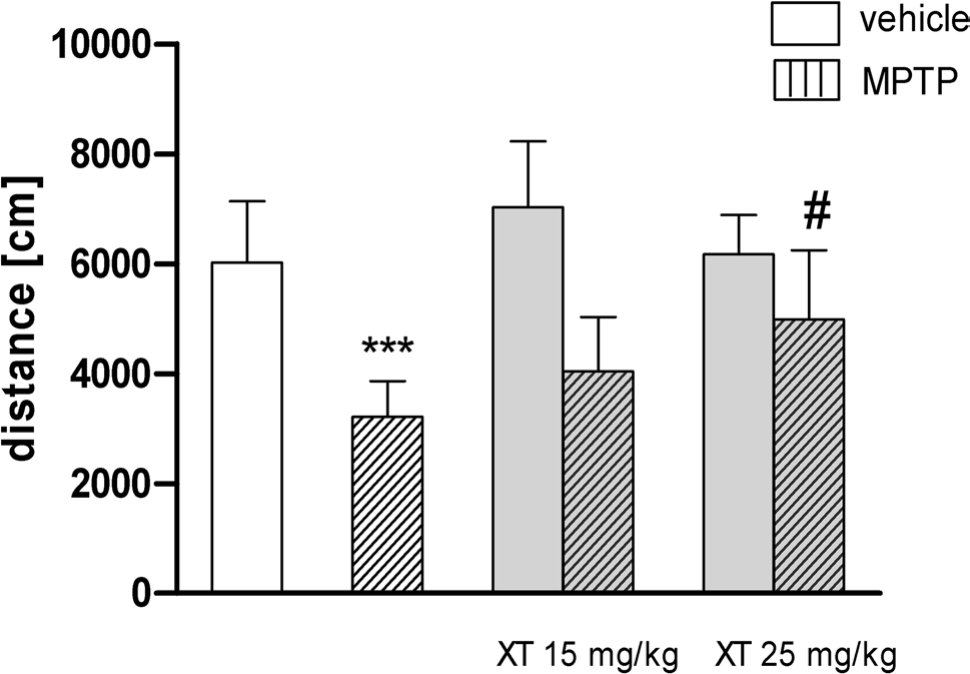
The effects of xanthotoxin (XT) on 1-methyl-4-phenyl-1,2,3,6-tetrahydropyridine (MPTP)-induced locomotor impairments. Mice were injected with saline or MPTP (20 mg/kg, 4 doses a day, every 2 h). XT (15 and 25 mg/kg) was administered acutely, immediately before locomotor activity measurement 14 days after MPTP administration. Data are presented as the mean ± SEM, ***p < 0.001 vs. saline control group; ^**#**^*p* < 0.05, vs. MPTP-treated group, *n* = 8; Bonferroni’s post hoc test

Figure 4 shows the distance traveled by mice after MPTP and XT treatment (two-way ANOVA: pretreatment (saline/MPTP) *F*_1,48_ = 66.98, *p* < 0.001; treatment (saline/coumarins) *F*_2,48_ = 3,94, *p* = 0.0261; and interaction *F*_2,48_ = 3.83, *p* = 0.0273. MPTP treatment (4 × 20 mg/kg) significantly decreased distance traveled by mice (*p* < 0.001) as compared to normal control group. Acute administration of XT (25 mg/kg) alleviates movement impairment induced by MPTP (*p* < 0.05); whereas, coumarin did not influence the locomotor activity in healthy mice in comparison with saline-treated control group (Fig. 4).

## Discussion

Our experiments for the first time evaluate the symptomatic properties of XT, a furanocoumarin widely occurring in the *Apiaceae* family, alleviating locomotor impairment induced by neurotoxins acting in the nigrostriatal system. Although many reports showed neuroprotective properties of coumarins, their bioactivity in contexts of PD was not evaluated using in vivo behavioral models till now. In this study, two different models employing zebrafish larvae treated with 6-OHDA and mice treated with MPTP were used. For the first time, two behavioral protocols were applied to evaluate and compare the potential of XT to reverse motoric symptoms of PD in animal models.

Zebrafish is a unique in vivo model with many advantages over rodents: a large number of offspring, more economic and easier housing and maintenance, the small size of larvae allowing to use big groups of animals in limited space of well plate and observing effects of a substance on a complex living organism. These features make zebrafish a perfect model for preliminary, screening investigations; hence, zebrafish was the animal model of choice in the first place [22]. Wide range of concentrations of XT has been investigated and after obtaining positive results on zebrafish treated with 6-OHDA, we decided to compare results with a different animal model and using a toxin that causes more drastic injuries in the nigrostriatal system (MPTP). 6-OHDA is widely used to cause dopaminergic degeneration both in rodent and fish model of PD. Administration of 6-OHDA results in motoric behavioral deficits and dopamine neuron losing the substantia nigra [23]. In zebrafish treatment with 6-OHDA decreased the number of DA neurons markedly in the diencephalon [24].

MPTP has been proved to be an important factor causing PD-like symptoms in both humans [25] and primates [26]. Repeated administration of MPTP causes the development of the parkinsonian features in mice [27]. In rodents, behavioral symptoms are accompanied by a significant decrease of the dopamine and its metabolites in the striatum with a small change in other neurotransmitters [28]. Knowing that MPTP causes severe neuronal damages in the CNS, we decided to use higher doses of XT than established in previous studies evaluating the influence of this compound on memory and anxiety-like behaviors in mice [8].

Both models confirmed that in higher doses, XT reversed PD symptoms. In our study, XT at the concentration of 7.5 µM reverses the locomotor reduction induced by 6-OHDA in zebrafish larvae. Additionally, single doses of XT (15 and 25 mg/kg) did not influence locomotor activity in mice; whereas, an acute dose of 25 mg/kg induced an increase in motor activity observed in the MPTP-treated mice. Many mechanisms may be responsible for observed effect. XT, as well as auraptene and daphnetin, may change the dopamine levels as MAOs or COMT inhibitor. Auraptene shows selective inhibitory effects against MAO-B at the concentration of 0.6 µM [29]. Daphnetin and its methylated metabolite (8-O-methyldaphnetin) are found to inhibit COMT with IC50 values 0.51–0.53 μM and 22.5–24.3 μM, respectively [30]. XT also may reverse locomotor impairment similar to scopoletin, which shows the antidepressant effects mediated by the activation of dopamine D1 and D2 receptors. This mechanism was confirmed by the use of a selective dopamine D1 receptor antagonist, SCH23390, and the dopamine D2 receptor antagonist sulpiride, both of which significantly antagonized the anti-immobility effect of scopoletin in the tail suspension test [31, 32]. Another simple coumarin—scoparone (100–200 µM) displays neuroprotective properties and enhances DA biosynthesis in PC12 cells [33].

In the previous studies, activity of two simple coumarins: umbelliferone and esculetin, was evaluated in MPTP-induced PD symptoms in mice, and both compounds showed neuroprotective activity. Umbelliferone and esculetin were administrated in the diet for 7 days at the dose of 0.75 mg/kg/day. The neuroprotective effect was measured as attenuation of the MPTP-induced decrease in TH neuronal staining in the substantia nigra pars compacta and both coumarins restored it to 75% of the control unexposed to the neurotoxin. Moreover, protection against MPTP-induced tyrosine nitrosylation, MPTP-induced decrease of glutathione level in the brain and MPTP-induced apoptosis were observed. The authors hypothesized that both coumarins were acting as antioxidants and, in consequence, reduced the oxidative and nitrosative damage caused by MPTP exposure [34]. Another simple coumarin with neuroprotective effects against MPP + -induced cytotoxicity is osthole. The compound enhanced the viability of rat pheochromocytoma PC12 cells exposed to MPTP at concentrations of 0.01, 0.05 or 0.6 mM reaching 32%, 44% and 51% neuroprotection, respectively [35]. In another study, anti-neurodegenerative potential of hydroalcoholic extract of *Ferulago angulata* (Schltdl.) Boiss, known for the content of furanocoumarins, e.g., XT, isopimpinellin, oxypeucedanin and oxypeucedanin hydrate [36], was examined against 6-OHDA PD symptoms in rats. Extract, after 14 days of oral administration, at the doses of 100, 200 and 400 mg/kg, significantly decreased lipid peroxidation in the striatum and hippocampus, a syndrome characteristic of PD [37, 38].

Since characteristic features of PD are high levels of basal oxidative stress markers in the substantia nigra, the important factors to prevent the disease progression are antioxidants [39]. Naturally occurring coumarins like esculetin, fraxetin, and daphnetin might affect the formation and scavenging of reactive oxygen species (ROS) [40]. XT shows moderate antioxidant properties according to the ferric-reducing antioxidant power (FRAP) and phosphomolybdenum-reducing antioxidant power (PRAP) [41]. It was revealed that the oxidative stress parameters altered by single and repeated injections of XT (1, 2.5 mg/kg) in mice showed protective properties. In this experiment, total antioxidant capacity (TAC) and concentration of malondialdehyde (MDA) in the hippocampus and prefrontal cortex were measured in mice. The first parameter can measure cellular defense against oxidative stress and the second one reflects the damages of lipids of the cells done by ROS. XT has not influenced on TAC but prevented the increase in MDA level induced by a single dose of scopoletin in the prefrontal cortex and hippocampus [8].

## Conclusions

In this study, the therapeutic potential against PD-like motor symptoms was evaluated. For this purpose, two different animal and pharmacological models were used. The studies confirmed that the zebrafish PD model and mice PD model are comparable. In both the models, we observed the locomotor activity impairments after administration of 6-OHDA and MPTP. Regardless of the pharmacological model, XT reversed the locomotor impairment induced by neurotoxins damaging nigrostriatal cells. Considering that XT belongs to coumarins, this effect may be a result of combined properties like antioxidative, inhibition of MAOs and COMT or agonistic effect on dopamine receptors. Although we are aware of the limitation of this study, it shed new light on neuropharmacological properties of XT and placed it as a potential lead compound in the discovery of antiparkinsonian drugs.

## Data Availability

The raw data supporting the conclusions of this manuscript will be made available by the authors, without undue reservation, to any qualified researcher.

## Abbreviations

6-OHDA: 6-Hydroxydopamine
ACh: Acetylcholine
AChE: Acetylcholinesterase
CNS: Central nervous system
COMT: Catechol-*O*-methyltransferase
CRF: Corticotropin-realizing factor
DA: Dopamine
HPCCC: High-performance counter-current chromatography
l-DOPA: Levodopa
MAO-A: Monoamine oxidase type A
MAO-B: Monoamine oxidase type B
MDA: Concentration of malondialdehyde
MPP+: 1-Methyl-4-phenylpyridinium
MPTP: 1-Methyl-4-phenyl-1,2,3,6-tetrahydropyridine
NMDA: *N*-Methyl-d-aspartate
PD: Parkinson’s Disease
TAC: Total antioxidant capacity
TH-IR: Tyrosine hydroxylase immunoreactive
XT: Xanthotoxin
